# Upper gastrointestinal endoscopy for dyspepsia: Εxploratory study of factors influencing patient compliance in Greece

**DOI:** 10.1186/1471-230X-11-11

**Published:** 2011-02-14

**Authors:** Eirini Oikonomidou, Foteini Anastasiou, Ioannis Pilpilidis, Elias Kouroumalis, Christos Lionis

**Affiliations:** 1 General Practitioner, Rural Setting Sindos, Health Centre Diabata, Thessaloniki, Greece; 2 General Practitioner Rural Setting Pyrgos, Health Centre Charaka, Crete, Greece; 3 Gastroenterologist, Theageneio Hospital of Thessaloniki, Greece; 4 MD, PhD, Professor, Department of Gastroenterology, School of Medicine, University of Crete, Heraklion, Crete, Greece; 5 MD, PhD, Hon FRCGP, Professor, Head of the Clinic of Social and Family Medicine, School of Medicine, University of Crete, Heraklion, Crete, Greece

## Abstract

**Background:**

Upper gastrointestinal endoscopy is the most preferable diagnostic examination for patients over fifty when upper gastrointestinal symptoms appear. However, limited knowledge exists in concerns to the compliance of primary care patients' to the doctors' recommendations for endoscopy.

**Methods:**

Patients who visited primary care practices in Greece and experienced upper gastrointestinal symptoms within a 10 days screening study, were referred for an upper endoscopy exam. The patients which refused to complete the endoscopy exam, were interviewed by the use of an open- ended translated and validated questionnaire, the Identification of Dyspepsia in General Population (IDGP) questionnaire. A qualitative thematic analysis grounded on the theory of planned behavior was performed to reveal the reasons for patients' refusal, while socio-demographic predictors were also assessed.

**Results:**

Nine hundred and ninety two patients were recorded, 159 of them (16%) were found positive for dyspepsia and gastro-esophageal reflux disease according to the IDGP questionnaire. Out of the above, 131 (83.6%) patients refused further investigation with endoscopy. Patients who refused upper endoscopy were predominantly female (87.8%) (*p *= 0.036) and over the age of 50. The lack of severe symptoms, fear of pain, concerns of sedation, comorbidity and competing life demands were reported by patients as barriers to performing an endoscopic investigation.

**Conclusions:**

Patients with dyspepsia in rural Greece tend to avoid upper gastrointestinal endoscopy, with two major axons considered to be the causes of patients' refusal: their beliefs towards endoscopy and their personal capability to cope with it. Future research examining reasons of low compliance should be carried out in combination with modern behavioral theories so as to investigate into the above.

## Background

Gastrointestinal disorders, in particular dyspepsia, are common problems within primary care worldwide, [1-3] as well as in Greece [4,5]. Experimental evidence on dyspepsia management is scarce and guidelines are based on information drawn from trials and clinical studies conducted in academic or specialist settings. It has been shown that their impact on general practices may be eminently critical in order to invalidate the implementation process [6]. Current guidelines suggest that all patients with dyspepsia over 45 or 55 years of age or those with symptoms should undergo prompt esophagogastroduodenoscopy (EGD) [7]. However, a successful implementation of recommendations that include invasive screening in everyday primary care practice seems to be related to factors such as the doctor-patient relationship and the patient's compliance to the doctor's recommendations [8,9].

For example, factors such as family history, perceived risk, self-efficacy, knowledge of the disease, or the use of educational videotaped material, were not proved to influence patients' decision about colorectal cancer (CRC) screening with a Faecal Occult Blood Test (FOBT) [8]. On the other hand, within primary care, compliance to colonoscopy and FOBT has been demonstrated to increase by the simple use of personalized encouraging brochures [9,10]. Therefore, explaining and modifying patients' attitudes in order to obtain higher compliance rates requires also a thorough knowledge of the factors that may influence their decision making process. Emphasis is given currently on patient centered communication and shared decision making that seem to lead to a significant increase in patient knowledge, improve quality of life and patient's satisfaction towards medical care, and also to reduce the anxiety and decisional conflict [10,11].

The human decision making process has been analysed thoroughly during the past decades and models have been developed that could explain the compliance of the patients towards the doctors' recommendations [11-13]. Various theories towards understanding and modifying human behaviour have been applied and among them the Theory of Planned Behaviour (TPB) has been constructed [14].

A PhD study that focussed on screening for upper gastrointestinal symptoms in a primary care population was designed and implemented into two Greek regions. Patients who visited selected rural practices were assessed; those who were positive for upper gastrointestinal symptoms were referred for upper endoscopy. This paper reports the findings of a qualitative study that was designed to reveal patients' reasons for refusing to undergo an endoscopy recommendation by their personal physicians within the use of the TPB.

## Methods

### Setting

**Figure 1:**
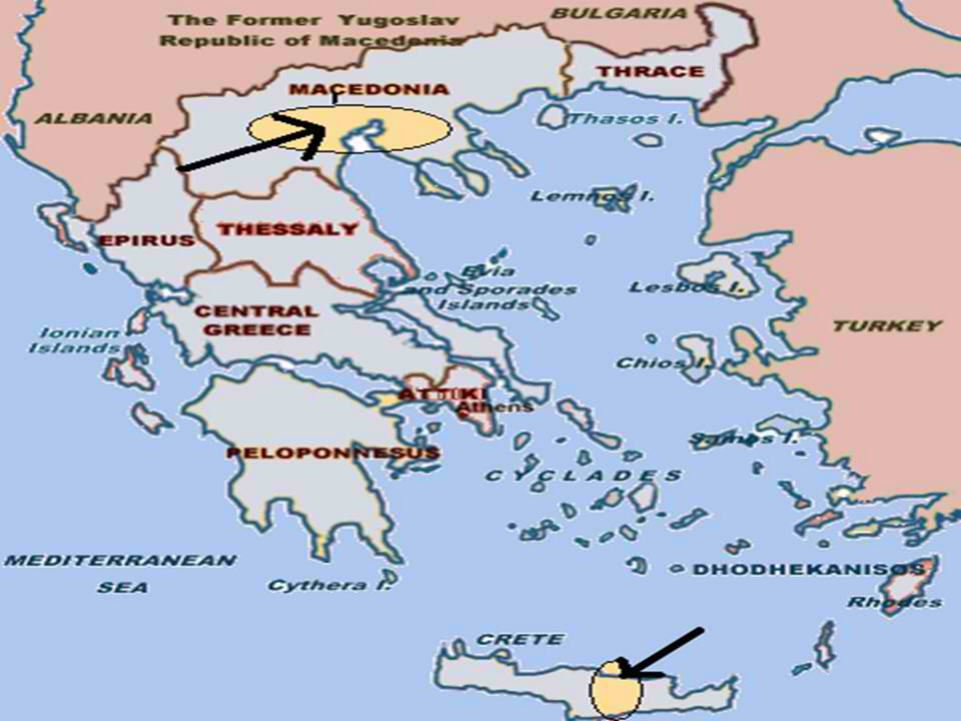
**Setting of the study**.

Five rural practices in Greece (three from the Greek region of Macedonia and two from the island of Crete), serving 21,100 residents in total were included in this study. The two areas differ only in terms of geography. The setting of the study is illustrated in Figure 1.

### Participants

All patients who visited the five rural practices within ten consecutive working days participated in the study (N = 992). The inclusion criteria were: over 18 years of age and patients without symptoms or a history of cancer and/or inflammatory bowel disease. The patients were residents of these specific areas within the rural settings.

Patients were assessed for upper gastrointestinal symptoms using a Greek translation of the validated "Identification of Dyspepsia in General Population" (IDPG) questionnaire named [4]. Patients who were found to be positive for dyspepsia or gastroesophageal reflux were referred to a gastroenterologist for further evaluation by EGD, within a ten day period with no financial burden. All patients refusing endoscopy were interviewed for the reasons of their denial.

### Interview development

A semi-structured interview consisting of three open-ended questions was used. The interview was guided by the TPB. The TPB supports that knowledge itself is not enough to lead to a certain action and that intention, together with perceived behavioral control, predict the likelihood of a person to actually perform a certain behavior. According to Ajzen [14], attitudes towards the behavior, subjective norms, and perceptions of behavioral control, are the three major determinants of the theory. These determinants are traced in the corresponding sets of behavior-related beliefs.

"Behavioural beliefs refer to the expected consequences of the planned behaviour, normative beliefs refer to the perceived behavioural expectations of important referent individuals or groups e.g. family, friends, while control beliefs have to do with the perceived presence of factors that can facilitate or impede the performance of a certain behavior." [14] This framework was utilized to explore patients' beliefs that lead to a certain intention and that intention to the denial of endoscopy.

### Questionnaire and interview

The questionnaire comprised of three questions; question 1 ("*What were your main reasons for denying EGD?"*) focused on the patients' negative attitudes towards endoscopy, question 2 ("*Is there any possible inconvenience in the performance of endoscopy?"*) evaluated the patients' control beliefs concerning their non-compliance to perform an endoscopy while question 3 ("*Do you think that the EGD that was suggested by your Family Physician, is important for you health?"*) presented mainly the perceived behavioural control and explored to what extent the physicians recommendations can facilitate patients behaviour. Interviews that took place in each rural setting were 10-15 minutes in length. Data concerning socio-demographic and other health issues for each patient were also recorded.

### Analysis of data

A qualitative content analysis was performed. Qualitative data were shorted and categorized by theme and this procedure was undertaken by the two principal investigators (EO, FA). They reviewed all the interviews and produced a consensus-coding document. Themes available in the TPB were used for the construction of the conceptual categories in our content analysis. In case of any inconsistency among the two reviewers, the issue was discussed and resolved [15,16].

### Ethics

The Scientific and Ethics Committee of the University Hospital of Crete approved the study (Number of protocol: 11873 - 25/10/2006). All participants received written information about the study's aim, the voluntary nature of participation and the assurance of confidentiality. All provided a signed consent form.

## Results

### Participants' characteristics

**Figure 2:**
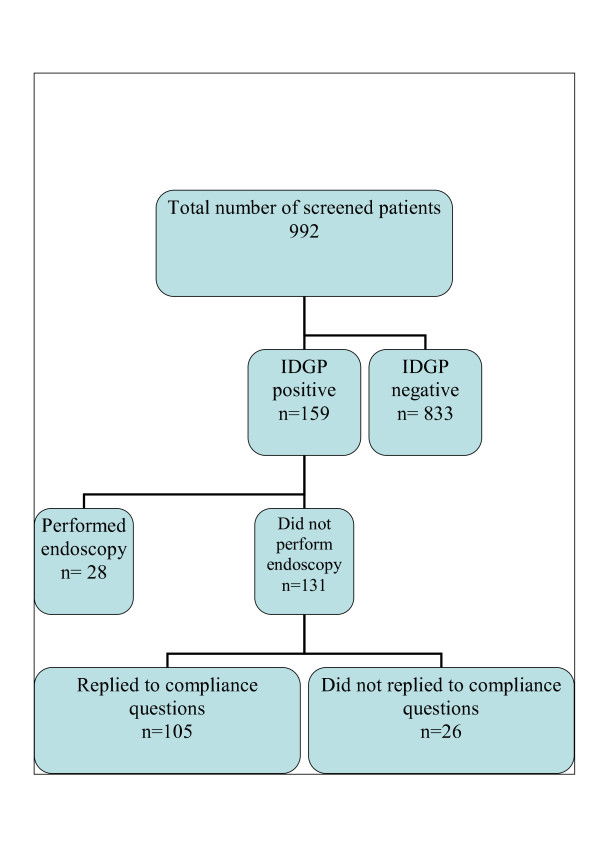
**Population of the study**.

According to the IDGP questionnaire, 159 patients were found positive with upper gastrointestinal symptoms, while 131 (83.6%) of them refused to proceed to an EGD. One hundred and eight (81.2%) were over 50 years of age. All non-compliants were interviewed by their participating General Practitioners (GP) yet 26 of them (19.8%) refused to answer the questionnaire. Figure 2 depicts the study population characteristics. The socio-demographic characteristics of the patients who proceeded for further evaluation with an EGD and the characteristics of patients who denied upper endoscopy are shown in Table 1. Male gender was the only socio-demographic factor that predicted a tendency of non adherence.

**Table 1:** Demographic Characteristics of Adherent and Nonadherent Patients to EGD

	Adherent patients (Ν = 28,17.6%)	Non Adherent patients (Ν = 131, 82.4%)	Statistical significance
**Sex**			
**Male**	5(10%)	45 (90%)	0.88
**Female**	23 (21.1%)	86 (78.9%)	
**Age**			
**> = 50**	25 (22.9%)	108 (77.1%)	NS
**<50**	3 (26.1%)	23 (73.9%)	
			
**Education**	**Primary school**	**High school**	**Higher**
**Adherent patients**	20 (16.9%)	6 (22.2%)	2 (7%)
**Non adherent patients**	98 (83.1%)	21 (77.8%)	10 (83.3%)
	NS	NS	NS
			
**Region**	**Central Macedonia, Greece**	**Crete, Greece**	
**Adherent patients**	19 (15.3%)	9 (25.7%)	
**Non adherent patients**	105 (84.7%)	26 (74.3%)	

Table 2 illustrates the number and the answers of the interviewed patients.

**Table 2:** Answers of interviewed patients who endorsed barriers to completing an Upperendoscopy

***Patients' main reason for denying endoscopy Question 1: ***What were your main reasons for denying EGD?		
*Barrier*	*Total (105)*	
External determinants	27	
		
Fear	24	
Not quite severe symptoms	21	
		
Difficult procedure	15	
		
Previous upper endoscopy	12	
		
I don't Know	6	
		
***Patients' possible inconvenience to endoscopy Question 2: ***Is there any possible inconvenience to the performance of endoscopy?		
*Barrier*	*Total (105)*	
Painful/difficult examination	41	
Fear	17	
External determinants	6	
PPIs use as an alternative	1	
		
Not inconvenient	30	
I don't know	10	
		
***Patients' beliefs concerning the importance of endoscopy for their health Question 3: ***Do you think that the EGD that was suggested by your Family Physician is important for you health?		
*Barrier*	*Total (105)*	
Important	41	
Possibly important	20	
		
Important/tendency to postpone	16	
PPIs use as an alternative	3	
Not important	19	
I don't know	6	

In order to avoid overlap across themes, the participants' responses were grouped and categorized according to the main barrier endorsed for refusing an upper endoscopy and are categorized by the TPB below.

### Patients' main reason for refusing endoscopy (behavioral beliefs)

#### Fear

A feeling of fear was the main reported obstacle in performing or even contemplating to undergo an EGD. Of the patients interviewed, 24 reported fear as a general feeling towards investigation (question 1).

*"I am afraid. I haven't been through it ever before." *(Patient 3, patient 102)

"I was afraid of what it could reveal. Let's better leave it to avoid worsening

*things." *(Patient 6)

Several patients (17) reported fear towards the procedure itself (question 2).

*"I think I am going to lose my breath and I could possibly die." *(Patient 33)

*"You could say that I am possibly afraid." *(Patient 75)

*".....Just the fear...." *(Patient 86)

### Perceived capability to perform the behavior (control beliefs)

#### Inconvenience

Patients (56) expressed both in question 1 and question 2 that endoscopy is a difficult, painful examination. They also focused on the way that it is performed.

*"I have been through a lot of exams but I have been informed that this one is very difficult and I decided not to do it." *(Patient 19)

*"I have heard that it is quite painful." *(Patient 63)

*"...well the fact that a whole tube is inserted through your mouth to your stomach."*(Patient 49)

#### Knowledge from self experience

Some participants (12) reported previous experience with EGD as a reason for not proceeding to a new one focusing again on the difficulty of the whole procedure (question 1). In several cases the diagnosis of the EGD which was reported orally by the patient was quite unclear.

*.......I almost died; they didn't give me any sedation, just a spray. I wouldn't like to be through it again*. (Patient 16)

#### No reason of inconvenience

A high percentage of the participants (30) reported no reason of inconvenience concerning the EGD (question 2).

*"No, nothing troubles me." *(Patient 107)

*"No, there is nothing to be afraid of. I would love to do it in the future." *(Patient 45)

#### Lack of knowledge

Some patients (16) declared uncertainty or they did not have a clear opinion of the EGD procedure (question 1 and 2).

*"I have never been through it before so I cannot tell you what makes me feel uncomfortable." *(Patient 19)

*"I cannot tell you since I don't know what it is about." *(Patient 64)

*"I haven't thought of it." *(Patient 99)

*"I really don't know." *(Patient 74)}

#### External determinants to the procedure

A high number of the participants (33) reported external determinants as an obstacle for not performing the EGD (question 1 and question 2).

"*I wouldn't like to put myself through trouble." *(Patient 49)

*"Somebody should escort me. I cannot move by myself."(*Patient 68)

### Patients' beliefs regarding the importance of the personal physician's recommendation to endoscopy (perceived behavioural control)

Patients' beliefs regarding the necessity and the importance of EGD for their health was questioned taking into account that endoscopy was recommended by their personal family physician.

#### Importance of an EGD examination

The participants (41) overall admitted the importance of EGD for their health.

*"Of course it is important." *(Patient 6)

*"Of course it is of great importance but you cannot force me to do it." *(Patient 26)

"*Very important if you take into account that I am suffering from my stomach almost every day." *(Patient 45)

In addition, many of them (20) replied that the procedure might be of importance. These were patients whose symptoms were not severe and mainly believed that they do not have a serious health problem.

*"Maybe, but one way or another I do not feel ill.*" (Patient 62)

*"I am not afraid of my problem with my stomach even though having an EGD might be important." *(Patient 51)

Some patients (16) showed a tendency to avoid or postpone the EGD, despite the fact that the examination could possibly be important for their health.

*"May be in the future but now I don't want to have it done." *(Patient 28) *"Yes, but I am going to have it done if I get worse." *(Patient 59)

Also, a few patients (4) stressed that Proton Pump Inhibitors is is always a preferred alternative solution (Question 1 and 3).

"*Well, as far as the job can be done with pills...Let's try that for the moment." *(Patient 1)

"*In general yes, but since it gets better with a pill, the exam is not that necessary*." (Patient 93)

A considerable part of participants (19) estimated that EGD, as an examination, is not important for the patient's health in general, and a few of them even questioned their personal doctor's opinion.

*"No it is not important. There are so few symptoms." *(Patient 107)

*"..so whatever the doctors say is always important?"(*Patient 22)

*"Many things are important but if we did all that the doctors say...." *(Patient 10)

Some patients (6) were unaware whether performing an EGD could be of any importance to their health.

## Discussion

### Main findings

This exploratory study highlighted the main reasons for patients' refusal to comply with upper gastrointestinal endoscopy. Even though patients seem to accept the necessity and the importance of an EGD for their health, they expressed certain barriers and fears towards the examination as their main reasons for noncompliance. Various factors that may influence adherence to screening tests (such as breast and colorectal cancer (CRC) screening) have been published in the literature [17-19]. With the fear of pain and fear of the procedure itself noted as important barriers for performing a mammography [17,19,20]. Studies concerning compliance to endoscopy for CRC screening revealed that the fear of pain, concerns and anxiety towards the procedure served the role of negative predictors for any form of CRC screening [18,21]. Similar fears and concerns were disclosed in this exploratory study.

The patient's level of education and knowledge of the procedure has been identified to play a critical role in adherence of the GP's instructions [22,23]. However knowledge has not been proven to be the only crucial factor to impel a certain action [14,24]. Almost 9% of the participating patients declared that additional data and explanation was needed, even though effort was made by the physicians (during consultation) to explain the need and the importance of endoscopy as well as describe the procedure itself. Many patients also reported that their own previous experience was either traumatic or not reassuring enough for them to proceed with a new endoscopy. Future intervention research should focus more on controlled beliefs and study their effectiveness on compliance rates to upper endoscopy.

Another interesting finding of our study was that the GP's recommendation for endoscopy (question 3) did not seem to facilitate patients to undertake the control of their behavior. It is known from the literature that advice, recommendation or encouragement from the personal physician can increase the likelihood of attendance of a certain examination [25-27]. Nevertheless in this study population, patients' perceptions seemed to play an important role as well, since 18% of the patients appeared to question their GPs' recommendation. Moreover, patients who undergo endoscopy generally report a larger number of, and more severe symptoms than those who do not [22]. Accordingly, upper gastrointestinal symptoms do not seem to have been considered as severe enough to obligate the patients to undergo an EGD.

Although no direct question in the interview focused on exploring normative beliefs and particularly to assess the extent that the patients' behaviour depends on their friends, family and the society to perform the recommended behavior, nevertheless some patients reported unpleasant experiences of individuals in their close environment as a main reason for avoiding the endoscopy.

Health problems and current life demands were the most common external determinants causing non compliance to EGD; yet when patients were asked in specific, for a reason of inconvenience to endoscopy these factors were impressively diminished. It seems that although undoubtedly important, these external barriers that were indicated during interviews were often vague and other obscure reasons for non adherence were referred which would need further investigation [18].

Different health insurance coverage seems to have a negative effect on the use of CRC tests [18,28]. However this was not the case in this study, since all EGDs were fully covered by the patients' insurance. The access to the referral centre and the long waiting times are other crucial factors of non adherence to recommendations but this factor cannot explain the high rate of non compliance in the study [17-19 ]. Strategies were utilized to overcome any waiting problems by scheduling the upper gastrointestinal endoscopy within a period of ten days. Access was not a main obstacle for the patients because there were no differences in compliance between the most and the less proximate to the hospital practices. In the TPB, demographic and personal characteristics of the patients may influence behaviour indirectly by affecting behavioural, normative, and control beliefs. The male gender was the only socio-demographic factor that predicted a tendency of non adherence in the population studied. This seems to be in controversy with other studies where female gender is the non compliance factor for CRC screening [18,28].

### Limitations

This study followed mainly a qualitative approach and consequently its findings cannot be extended to wider populations. Also, the population studied poorly represented minorities and was not socioeconomically diverse. However, its low adherence suggests that this is likely to be an even greater problem among less socially advantaged patients. Patient-level influences on adherence have been determined, through interviews on a small sample of patients. Consequently the results are not strong enough to permit us to draw firm conclusions. The interviews reflect only the perceptions of patients who refused endoscopy and cannot be compared with the perceptions of patients who had undergone endoscopy.

Nevertheless, this exploratory approach was warranted because no prior studies have approached the causes of refusal of upper gastrointestinal endoscopy. The number of the interviewed subjects was higher than that in usual qualitative studies, due to the design of the study which was initially a part of quantitative research, and at a second stage we decided to focus more on an exploratory qualitative direction.

The TPB was successfully utilized to explain patient's answers. Compared to affective processing models, the theory overlooks emotional variables such as threat, fear, mood and negative or positive feeling and assesses them in a limited fashion. In the health related behavior situation, given that most individuals' health behaviors are influenced by personal emotion and affect, this could be a drawback for predicting health-related behaviors [29].

## Conclusion

This study provides the first insight towards understanding primary care patients' reasons for denying upper gastrointestinal investigation. It is clear that neither expert's opinion nor adherence to guidelines is enough to persuade patients to follow a procedure such as EGD. There are still some uncertainties regarding the factors that affect patients' behavior, however, it remains to be examined whether incorporated multifaceted interventions that will explore the multiple barriers encountered in primary care, might improve adherence to upper endoscopy. The study findings are of interest to both GP/Family Physicians and health policy makers, who need to focus more on the doctor-patient communication issues and the cultural setting where the primary care services are provided, which can have an impact on the early referral of patients with dyspepsia and the early diagnosis of severe diseases.

## List of abbreviations used

EGD: Esophagogastroduodenoscopy; FOBT: Fecal Occult Blood Test; GP: General Practitioner; CRC: Colorectal Cancer.

## Competing interests

The authors declare that they have no competing interests.

## Authors' contributions

EO was the principal investigator for the study and undertook data collection, transcribed and analysed the interviews, and wrote the initial manuscript and revised it upon the editor's and reviewers' guidance. FA undertook data collection and took part in the qualitative analysis of the interviews. IP and HC contributed to the study design and gave comments on the manuscript. CL conceived the initial idea, contributed to each stage of the study development, analysis, reporting, reviewed the analysis and interpretation of data, corrected the first draft, co-designed the contents and revised together with EO the manuscript. Members of the Greek General practice Dyspepsia Group* have made substantial contributions to acquisition of data. All authors have participated in the design of the study and have commented critically on the initial manuscript and have approved the final version of the manuscript.

*Greek General Practice Dyspepsia Group: Androniki Glystra (aglystra@yahoo.gr), Sofia Dimopoulou (dimosophie@yahoo.gr), Ioanna Tsiligianni (pdkapa@yahoo.gr).

## Pre-publication history

The pre-publication history for this paper can be accessed here:

http://www.biomedcentral.com/1471-230X/11/11/prepub
